# Measurement of chemical information using 3D ED and Cryo-EM

**DOI:** 10.1063/4.0000841

**Published:** 2025-10-27

**Authors:** Koji Yonekura

**Affiliations:** RIKEN, Sayo, Hyogo, Japan. Tohoku University, Sendai, Miyagi, Japan

## Abstract

While single-particle cryo-EM involves freezing protein solutions, eliminating the need for crystallization, it is limited by the molecular weight of the sample due to image contrast. In contrast, electron crystallography, particularly 3D electron diffraction (3D ED) or MicroED, enables high- resolution structural analysis from small crystals that are undersized for conventional single-crystal X-ray diffraction. This technique is increasingly being applied to a wide range of samples, from small proteins and polypeptides to low- and medium-molecular-weight compounds relevant to drug discovery and materials science.

We are particularly interested in extracting detailed chemical information using 3D ED and single-particle analysis (SPA) and have been pursuing research and development in this area. While X-rays are scattered by electrons surrounding atomic nuclei, electron beams interact with the Coulomb potential along their transmission path, exhibiting significantly higher sensitivity to valence electrons. Leveraging this property, we have recently reported the visualization of charge distributions and the measurement of chemical bonds using high-resolution SPA and 3D ED.

In SPA, we demonstrated that high-resolution structure analysis of a protein complex can distinguish different types of hydrogen bonding and reveal extract charge-related information. Hydrogen atoms, charge distributions, and chemical bond polarity in proteins play crucial roles in structural stabilization, enzymatic catalysis, energy transfer, and interactions with substrates or drugs. However, experimental measurement of these properties remains challenging. Our findings suggest that single-particle analysis can contribute to a deeper understanding of the chemical properties and functions of biological macromolecules.

In 3D ED, our group has been actively involved in the development of this technique, which now enables the rapid processing of large datasets through AI control, facilitating efficient structure determination. We have worked on a variety of samples, ranging from proteins to small molecules, including wild-type and mutant polypeptides associated with amyotrophic lateral sclerosis (ALS), the stereochemical configuration of functional groups in natural organic compounds used as pesticide candidates, the double-helix structure of nanographene, and the structural properties of newly synthesized organic semiconductors. However, this method has limitations related to crystal thickness and data quality. To address these challenges, we have developed serial crystallography for organic compounds using X-ray free electron lasers (XFELs) and demonstrated that combining these two techniques is highly effective in analyzing challenging targets.

In this symposium, I will introduce our approach and recent results, while also discussing the limitations and future prospects of 3D ED in comparison with microcrystallography using X-ray free electron lasers and single-particle cryo-EM.

